# Disrupted normal ingestion during glucose intake modulates glucose kinetics in humans

**DOI:** 10.1186/s40064-015-1419-3

**Published:** 2015-10-17

**Authors:** Tadataka Tsuji, Susumu Tanaka, Kumiko Kida, Sanam Bakhshishayan, Mikihiko Kogo, Takashi Yamamoto

**Affiliations:** First Department of Oral and Maxillofacial Surgery, Graduate School of Dentistry, Osaka University, 1-8 Yamadaoka, Suita City, Osaka 565-0871 Japan; Department of Health and Nutrition, Faculty of Health Science, Kio University, Nara, 635-0832 Japan

**Keywords:** Chemical senses, Blood glucose, Insulin, Salivary α-amylase

## Abstract

This study aims to reveal the importance of chemical senses in glucose kinetics and autonomic nervous activity by imposing interventions during glucose intake. The glucose-loading test was applied to seven healthy individuals. Three successive oral glucose-loadings induced a gradual downward shift in the blood glucose curves (BGC) together with increased salivary α-amylase activity (s-AMY) and positively correlated with satisfaction scores. On the other hands, adding a pleasant flavor given during the third trial increased the BGC to the same level as that during the first loading with decreased s-AMY value. Direct intragastric delivery of glucose or clipping the nose induced a downward shift in both BGC and serum insulin response curves (IRC), resulting in a decrease of the area under the BGC, positively correlated with the area under the IRC and satisfaction scores, respectively. The present study suggests that disrupted normal ingestion during glucose intake modulates glucose kinetics along with increased s-AMY values, indicating enhanced sympathetic nervous activity and favorable chemical senses are important in maintaining glucose kinetics.

## Background

Blood glucose level (BGL) is regulated by mechanisms of metabolic homeostasis (Leto and Saltiel 2012). Under normal circumstances, BGL is decreased by energy expenditure during exercise and cellular activities, and increased by various factors including food ingestion. Physiological glucose metabolism is mainly regulated by antagonistic types of metabolic hormones: anabolic hormones such as insulin that reduce BGL and catabolic hormones including glucagon, adrenaline and noradrenaline that increase BGL. Glucose intake during meals is the most efficient way of increasing BGL, which induces the secretion of insulin that in turn causes BGL to gradually decline to baseline levels after meals (Leto and Saltiel 2012). Insulin promotes the storage of reserve glucose in the liver as glycogen, suppresses both glycolysis and hepatic gluconeogenesis, forces glucose in the bloodstream to be taken into skeletal muscle and fatty tissues through the glucose transporter 4 (Bryant et al. 2002), and directly suppresses the secretion of glucagon (Leto and Saltiel 2012). Gluconeogenesis and glycolysis including the secretion of these hormones are also modulated by the autonomic nervous system (ANS) to maintain metabolic homeostasis (Corssmit et al. 2001).

These findings indicate that many factors are involved in modulating BGL during meals. Sensory factors such as taste, smell and texture during mastication in the oral cavity, food palatability (pleasant and unpleasant), mode of consumption, environmental factors and stress might affect digestive and absorptive functions of the gastrointestinal system through the autonomic nervous system, resulting in BGL modulation (Bravo 1989). Thus, the increase in BGL induced by meals and the post-prandial decrease in BGL are modulated by various factors comprising glucose absorption from the gastrointestinal tract, glucose uptake into the skeletal muscle and adipose tissues, glycolysis and hepatic gluconeogenesis, and autonomic nervous activity. However, how these factors modulate BGL during ingestive behavior remains unknown.

Tastes and odors, which are collectively called chemical senses, play important roles not only in constructing food palatability and triggering the desire to eat (Shepherd 2006), but also in influencing the autonomic nervous system (Kitamura et al. 2010). Oral ingestion stimulates chemical senses and increases insulin release including the cephalic phase (Abdallah et al. 1997) together with a pleasant feeling, resulting in BGL elevation followed by glucose uptake into the skeletal muscle via the autonomic nervous system. Palatability which is associated with these chemical senses might also play important roles in glucose metabolism through the secretion of such hormones and the autonomic nervous system. However, whether or not palatability affects hyperglycemic responses after meals in humans together with the autonomic nervous activity remains unknown.

To examine the importance of palatability, which is based on the chemical senses, in glucose kinetics and autonomic nervous activity, we imposed some interventions during glucose intake. For this purpose, the conventional glucose-loading test was used under various experimental conditions together with a self-reporting questionnaire about satisfaction. We then analyzed blood glucose curves (BGC), serum insulin response curves (IRC), and salivary α-amylase (s-AMY) activity and administering a questionnaire about satisfaction. Previous studies have shown that s-AMY activity is a useful marker of sympathetic nervous activity (Yamaguchi et al. 2004). We discuss that the hyperglycemic response after glucose-loading is dependent not only on the volume of loaded glucose but also on palatability and the mode of glucose administration.

## Methods

### Participants

Seven healthy individuals [male, n = 5; female, n = 2; mean age ± SE, 29 ± 0.7 years; mean body mass index (BMI) ± SE, 22.3 ± 1.1 kg/m^2^] were recruited from our medical staff. Four of the men (age, 27 ± 1.2 y; BMI, 21.7 ± 0.4 kg/m^2^) participated in both Experiments 1 and 2, and one man and the two women participated only in Experiment 2 (Table 1). A physical examination showed that all of them were in good health and none had a history of gastrointestinal or endocrine disorders. There were no family histories of diabetes which can potentially decrease the beta-cell response during the glucose loading. The study purpose and procedures were explained to the recruits, who each provided written informed consent to participate. The Ethics Committee of Osaka University approved the study (No. H19-E9-2), which proceeded in accordance with the ethical standards for human experimentation included in the Declaration of Helsinki.

**Table 1:** Basic information of participants

	Experiment 1	Experiment 2
Participants	4	7
Gender	Male	Male (5) and female (2)
Age	27 ± 1.2	29 ± 0.7
Body weight (kg)	63.2 ± 1.8	67 ± 4.4
Height (m)	1.70 ± 0.01	1.72 ± 0.03
Body mass index (kg/m^2^)	21.7 ± 0.4	22.3 ± 1.1
Resting glucose (mg/dl)	79.7 ± 2.1	79.6 ± 2.9
Resting insulin level (µU/ml)	–	5.5 ± 1.0

Data are shown as mean ± SE

### Procedure

#### Blood glucose levels and serum immunoreactive insulin

The experiment started at 9:00 am in a quiet room to avoid potential circadian and environmental effects. The participants did not consume food or liquids other than water for 12 h before, and cleaned their teeth and rinsed their mouths 15 min before starting the experiment. Each participant was fitted with an intravenous cannula (22 gauge; inside diameter, 0.80 mm; length, 32 mm; Terumo, Tokyo, Japan) for blood sampling. The first 5 mL of sampled blood was discarded to eliminate residual saline in the cannula. We initially measured baseline BGL before glucose-loading. Thereafter, 6 min of loading with a glucose solution (Glucose 75 g/225 mL, TRELAN-G75^®^; Ajinomoto Inc., Tokyo, Japan) randomly proceeded under the following conditions on the different days. We took a week interval as a recovery period between each trial. Then the three successive oral glucose-loading procedures in Experiment 1 lasted about 7 h (2-h-loading × 3 + 20-min-interval × 2), whereas single oral glucose-loading in Experiment 2 lasted about 2 h.

##### Experiment 1

*Trial 1* Three successive oral loadings of glucose at intervals of 140 min after the previous loading.

*Trial 2* Two successive oral loadings of glucose plus one oral loading of glucose with an agreeable aroma (selected by each participant from a choice of vanilla, milk, strawberry, pineapple and lemon).

##### Experiment 2

*Trial 1* A single oral loading of glucose.

*Trial 2* A single oral loading of glucose with the nose clipped to interfere with normal nasal air-flow.

*Trial 3* A single intragastric loading of glucose via a nasogastric tube to eliminate oral sensory information.

Blood samples (2 and 5 mL for Experiment 1 and 2, respectively) were collected at 15, 30, 45, 60, 90, and 120 min after each loading to measure plasma glucose and serum insulin. We firstly checked the blood glucose level using a GR-102 glucose meter (Terumo) and separated the blood plasma from 2 mL of whole blood by centrifugation at 3000 rpm for 10 min at room temperature. Then we measured the plasma glucose level utilizing the HK-G-6-PDH method in both Experiments. Glucose was measured twice at each time point to minimize systemic error. In Experiment 2, the serum separated from 3 mL of blood samples was transferred to chilled polypropylene tubes and stored at −60 °C. Immunoreactive insulin (IRI) was measured utilizing the ARCHITECT^®^ insulin chemiluminescence immunoassay kit (Abbott Laboratories, Tokyo, Japan) and an ARCHITECT^®^ analyzer *i*2000 (Abbott Laboratories). We applied an area under blood glucose curve (AUBGC) and serum immunoreactive insulin curve (AUIRC) from the start of the experiment until 120 min thereafter to the Eq. 1.

1 $${\text{AUC}}{ \,\fallingdotseq\, }\left( {{\text{Y}}_{ - 6} + {\text{Y}}_{ 1 5} } \right) \, \times \, \left( { 6 { } + { 15}} \right)/ 2 { } + \, \left( {{\text{Y}}_{ 1 5} + {\text{Y}}_{ 30} \times { 2} + {\text{Y}}_{ 4 5} \times { 2} + {\text{Y}}_{ 60} } \right) \, \times { 15}/ 2 { } + \, \left( {{\text{Y}}_{ 60} + {\text{Y}}_{ 90} \times { 2 } + {\text{Y}}_{ 1 20} } \right) \, \times { 3}0/ 2$$

Y_x_ is a BGL or IRI at the time point (x).

### Measurement of salivary α-amylase activity

Prior to the experiments, we evaluated the intra-assay coefficient of variation (CV) of salivary amylase activity measured with a device (COCORO meter, NIPRO Co., Osaka, Japan) (Yamaguchi et al. 2004). The intra-assay CV of ten successive saliva samplings of a participant was 5 % (average s-AMY: 45.5, SD: 2.17). We also examined the inter-assay CV for saliva samplings of four participants on three different days. Inter-assay CV was 10 % (average s-AMY: 43.7, SD: 4.52), indicating that the device is useful for salivary amylase measurement.

Saliva was sampled before (pre-) and immediately after (post-) glucose loading in Experiments 1 and 2, and 60 min after glucose loading in Experiment 2. Concentrations of s-AMY were measured using a simplified COCORO meter. Briefly, a disposable probe was inserted into the sublingual region for 30 s, and then s-AMY activity was measured for 10 s using the kit. Then the s-AMY ratio was defined as the value after glucose-loading when the s-AMY value before loading was taken as 1.0.

### Questionnaire about satisfaction after glucose loading

A self-reporting survey based on the visual analog scale (VAS) was distributed to the participants to determine levels of satisfaction after each glucose-loading. The VAS consisted of a horizontal line with one anchor point at each extreme (Grant et al. 1999). The descriptions “no satisfaction” and “sufficient satisfaction” were placed at the left and right ends of the scale, respectively. The VAS was scored from 0 to 10, but the participants were unaware of these values.

### Data analysis and statistics

The data in both experiments are presented as mean ± SE, median, and range (Tables 2, 3, and 4) because of small sample size. The time course such as the plasma glucose and the serum insulin level for 2 h among different conditions were assessed using a two-way repeated-measures ANOVA, followed by Scheffe’s test. Comparison of the AUBGC on successive loadings in Experiment 1 was assessed using Kruskal–Wallis test, whereas the AUBGC between conditions in Experiment 2 were statistically compared using a paired t-test. The s-AMY before and after glucose-loading within same participants was also statistically compared using a paired t-test or Wilcoxon signed-rank test. Correlations between the AUBGC, AUIRC, s-AMY ratio and satisfaction scores were assessed using Spearman’s rank-correlation coefficient tests. Differences were considered significant at *p* < 0.05.

**Table 2:** Time course of changes in blood glucose level and salivary α-amylase activities after successive glucose-loadings

			Blood glucose level (mg/dl)							AUBGC	s-AMY	
			Pre-loading	15 min	30 min	45 min	60 min	90 min	120 min	(mg/dl × min)	Pre-loading	Post-loading
Trial 1	1st GL	Mean ± SE	79.8 ± 2.1	121.5 ± 9.3	136.5 ± 12.8	150.5 ± 14.4	117.8 ± 12.4	98.5 ± 1.6	92 ± 3.4	14,314 ± 584	44.3 ± 3.7	41.5* ± 3.9
		Median	79.5	119	132.5	157.5	112	99.5	89.5	13,922	41.5	40
		Inter-quartile range	4.75	18.5	31.5	34.5	28.75	2.5	4.5	1108	6.3	7.5
	2nd GL	Mean ± SE	59.3 ± 5.5	109.5 ± 7.6	109.8 ± 10.7	107.8 ± 4.2	105.5 ± 2.3	81.5 ± 7.8	75.3 ± 0.9	11,803 ± 225	47.8 ± 3.4	45.3 ± 3.8
		Median	58	115.5	109	106.5	104	84.5	75.5	11,709	45	42.5
		Inter-quartile range	16.25	10.5	21.75	7.25	4	18.5	1.75	327	3.8	7.8
	3rd GL	Mean ± SE	75 ± 0.7	83.8 ± 3.8	95.3 ± 8.2	75.3 ± 9.8	96.3 ± 4.7	85.5 ± 3.2	80.5 ± 5.5	10,791 ± 448	53.3 ± 3.8	55.8* ± 3.2
		Median	75.5	80	102	69.5	96	84.5	75	10,685	52.5	55
		Inter-quartile range	1.5	3.75	13.25	23.25	7.25	6	5.5	884	8.3	6.8
Trial 2	1st GL	Mean ± SE	81.8 ± 1.9	129 ± 13.9	127 ± 9.4	126.8 ± 9.5	122.3 ± 6.2	101.8 ± 5.2	95 ± 7.3	14,215 ± 618	45.5 ± 2.1	41.8* ± 2
		Median	81.5	135.5	128	121	125.5	98	89.5	14,045	45	42.5
		Inter-quartile range	5.75	30	21.5	15.25	12.25	7.25	14.5	1145	6.5	5.8
	2nd GL	Mean ± SE	71.8 ± 7	125 ± 5.3	113 ± 9.8	108.3 ± 11.8	107.3 ± 5.8	89.3 ± 5.6	89.8 ± 5.1	12,759 ± 326	48 ± 2.4	47.8 ± 4
		Median	75.0	128.5	113.5	105.5	108.5	86.5	90.5	12,486	46	46.5
		Inter-quartile range	8.25	11	18.5	22.25	7.75	8.75	16.25	462	4.0	9.8
	3rd GL + pleasant odor	Mean ± SE	75.8 ± 8.7	121.5 ± 3.7	138.8 ± 6.8	126.8 ± 7.3	118 ± 9.9	105 ± 7.7	97.3 ± 5.1	14,229 ± 568	51.3 ± 4.1	48.8* ± 4.2
		Median	73.5	121	135.5	125	115.5	107	97	14,214	49	47.5
		Inter-quartile range	14.25	10	8.75	22.25	24	24	7.75	1879	6.3	5.8

Trial 1: three successive oral loadings of glucose at intervals of 140 min after the previous loading. Trial 2: two successive oral loadings of glucose plus one oral loading of glucose with an agreeable aroma

*AUBGC* area under the curve of blood glucose from starting to 120 min, *GL* glucose-loading, *s-AMY* salivary α-amylase

* *P* < 0.05 (after vs. before glucose-loading), paired t-test; n = 4

**Table 3:** Time course of changes in blood glucose level and salivary α-amylase activities after the different glucose-loadings

		Blood glucose level (mg/dl)							AUBGC	s-AMY		
		Pre-loading	15 min	30 min	45 min	60 min	90 min	120 min	(mg/dl × min)	Pre-loading	Post-loading	60 min
Control	Mean ± SE	79.6 ± 3.0	128.4 ± 6.6	129.9 ± 8.9	130 ± 9.6	121.5 ± 10.6	105.9 ± 5.8	92 ± 5.1	14,335 ± 692	45.3 ± 4.3	42.9 ± 3.5	40.1 ± 2.4
	Median	75	131.5	135.5	130.5	124	105	97	13,633	43	42	39
	Inter-quartile range	11.8	21.0	35.0	35.3	20.5	10.5	12.8	1776	10.5	8.5	5.5
Nose clipping	Mean ± SE	79.8 ± 1.7	117.9 ± 4.7	118.2 ± 11.3	111.1 ± 12.2	107.2 ± 9.2	93.9 ± 7	80.1 ± 3.8	12,832 ± 745	42.3 ± 1.8	48.1^†^ ± 2.2	42.1^#^ ± 0.9
	Median	78	122	128.5	96	106	93	80	12,607	42	47	41
	Inter-quartile range	6.3	13.3	50.0	56.3	39.8	26.3	13.0	2664	3.5	6.5	3.5
Intragastric	Mean ± SE	80.8 ± 1.6	115.4 ± 5.2	113.4 ± 11.8	104.6 ± 9.6	98 ± 8.1	87.9 ± 3.9	86.3 ± 3.9	12,335* ± 655	43.1 ± 4.1	49^†^ ± 4.2	44.7 ± 3.5
	Median	80.5	120	123	115	100	92	86.5	12,845	38	47	40
	Inter-quartile range	5.8	12.8	31.5	39.0	27.3	15.5	7.5	2198	10.5	9.0	6.5

*AUBGC* area under the curve of blood glucose from starting to 120 min, *s-AMY* salivary α-amylase

* *P* < 0.05 (intragastric vs. control), paired t-test; ^†^ *P* < 0.05 (post- vs. pre-loading) and ^#^ *P* < 0.05 (60 min vs. post-loading), Wilcoxon signed-rank test; n = 7

**Table 4:** Time course of changes in serum immunoreactive insulin level after the different glucose-loadings

		Serum immunoreactive insulin level (µU/ml)							AUIRC
		Pre-loading	15 min	30 min	45 min	60 min	90 min	120 min	(µU/ml × min)
Control	Mean ± SE	5.5 ± 1.0	43.1 ± 8.0	46.3 ± 8.9	46.5 ± 9.4	45.8 ± 9.9	35.6 ± 6.1	24.1 ± 3.9	4684 ± 725
	Median	5.1	40.7	47.1	38.8	44.6	39.6	23.2	4452
	Inter-quartile range	3.8	22.3	22.1	37.0	40.8	14.6	16.2	2764
Nose clipping	Mean ± SE	6.9 ± 1.3	35.5 ± 4.2	38.4 ± 7.2	37.5 ± 7.9	37.1 ± 8.3	23.6 ± 4.9	13.5 ± 2.7	3595 ± 481
	Median	7.5	31.7	37.7	26.3	34.5	20.0	15.6	3542
	Inter-quartile range	4.2	10.1	24.0	25.7	25.1	17.3	9.1	1360
Intragastric	Mean ± SE	6.7 ± 0.3	36.5 ± 5.2	37.2 ± 4.5	41 ± 7.6	37.1 ± 7	22.8 ± 3.4	16.6 ± 2.9	3668 ± 479
	Median	6.7	32.9	38.6	37.3	35.9	21.8	16.4	3774
	Inter-quartile range	0.5	16.5	8.2	24.3	17.2	8.8	7.0	1265

N = 7

*AUIRC* area under curve of time-course change in serum immunoreactive insulin level from starting to 120 min

## Results

### Experiment 1: successive oral glucose-loadings

As shown in Table 2, successive oral loadings of glucose in the trial 1 produced a downward shift of BGC in the order of the first to the third loadings [main effect of loading times; *F* (2, 54) = 18.46, *P* = 0.0007, main effect of time course; *F* (6, 54) = 15.78, *P* < 0.001, significant interaction between loading times × time course; *F* (12, 54) = 3.40, *P* = 0.001]. However, adding an agreeable flavor to the glucose solution at the third loading in trial 2 increased the BGC to a level comparable to that of the first loading. These results became more obvious when the AUBGC was calculated. Table 2 shows that the AUBGC gradually decreased from the first to the third loadings (*P* = 0.015), whereas the AUBGC of the third loading with an agreeable flavor was essentially the same as that of the first loading. Values for s-AMY at post-, compared with pre-loading were lower (*P* < 0.05) for the first loading, higher (*P* < 0.05) for the third loading in trial 1, and lower (*P* < 0.05) in trial 2 when the flavor of the glucose solution was agreeable (Table 2).

**Fig. 1:**
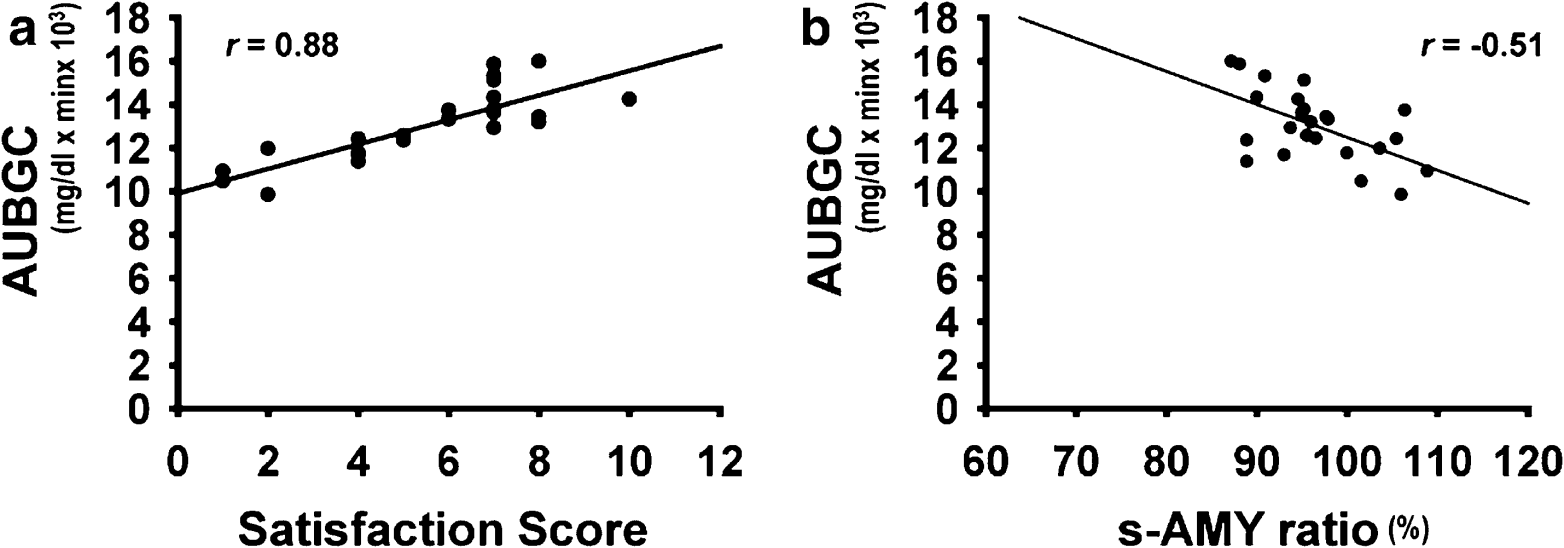
Correlations among area under blood glucose curves, salivary α-amylase activity and satisfaction scores. **a** AUBGC correlates with satisfaction score (*P* < 0.001); **b** post-loading s-AMY expressed as ratio of pre-loading value negatively correlates with AUBGC (*P* = 0.015). N = 24, Spearman’s rank-correlation coefficient tests. *AUBGC* area under blood glucose curve, *s-AMY* salivary α-amylase

Figure 1a shows a significant positive correlation (*r* = 0.88, *P* < 0.001) between AUBGC and satisfaction scores, indicating that the downward shift of the BGC closely corresponded to a decrease in satisfaction. The AUBGC and s-AMY ratio negatively correlated (*r* = −0.51, *P* = 0.015), indicating that AUBGC decreased as s-AMY increased (Fig. 1b).

### Experiment 2: effects of nose clipping and intragastric glucose loading

**Fig. 2:**
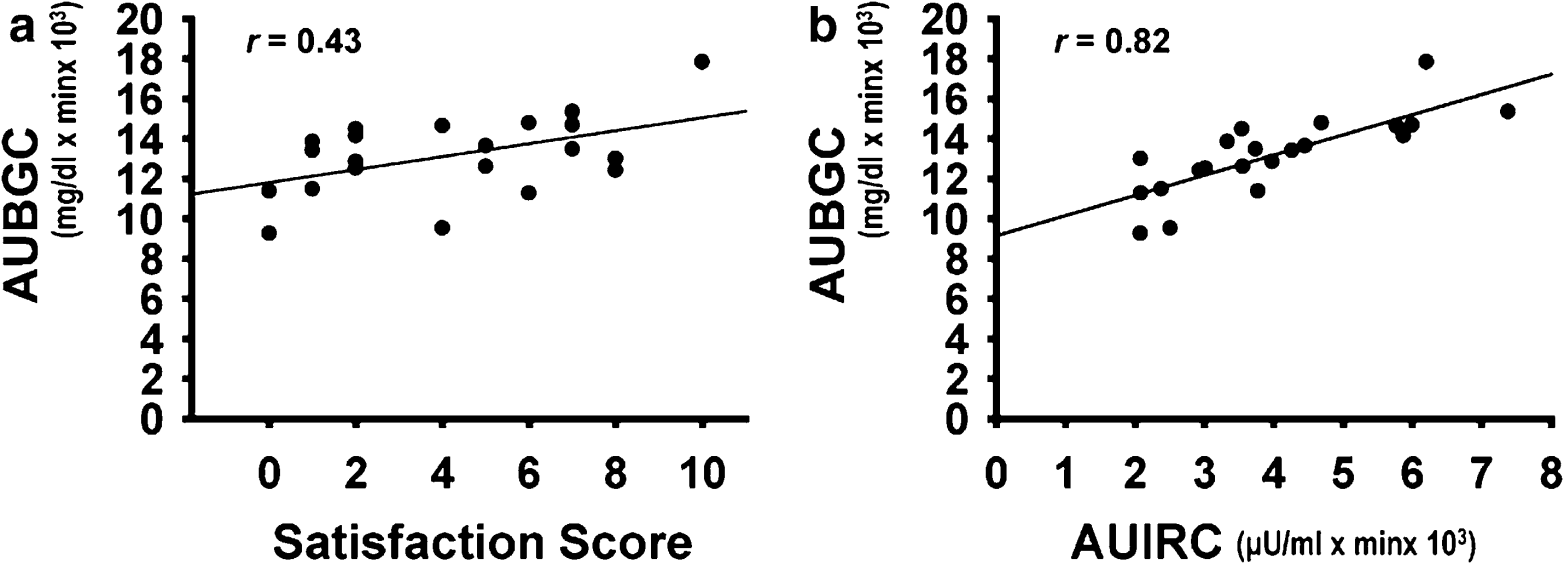
Correlations among satisfaction scores, area under blood glucose curves and serum immunoreactive insulin curves. **a** AUBGC correlates with satisfaction score (*P* = 0.05). **b** AUBGC correlates with area under curve of time-course change in IRI (AUIRC) (*P* = 0.0002). N = 21, Spearman’s rank-correlation coefficient tests. *AUBGC* area under blood glucose curve, *AUIRC* curve of changes in IRI, *BGL* blood glucose level, *IRI* serum immunoreactive insulin

Oral glucose loading with the nose clipped and intragastric glucose loading also produced a downward shift in BGC, which led to a decrease in the AUBGC (nose clipped and intragastric loading vs. control, *P* = 0.07 and *P* < 0.05, respectively; Table 3). These procedures also shifted the IRC downward (Table 4). Nose clipping increased (*P* < 0.05) the values of s-AMY after glucose loading, and then decreased (*P* < 0.05) 60 min later. Values of s-AMY under the intragastric loading condition also increased (*P* < 0.05) after glucose loading. Thus the s-AMY ratio (post-/pre-loading) significantly (*P* = 0.018) increased after nose clipping and intragastric loading. As shown in Fig. 2, the AUBGC positively correlated with satisfaction scores (*r* = 0.43, *P* = 0.05) and AUIRC (*r* = 0.82, *P* = 0.0002).

## Discussion

We have to admit that the sampling number (n = 4) in Experiment 1 was small. This was mainly because the three successive oral glucose-loading procedures lasting over 7 h was so stressful that we had a difficulty in recruiting enough participants. However we found that there were no large differences among the individual variabilities despite this small sample size (see Table 2), and could evaluate acquired data using the appropriate statistic procedures. To increase the data accuracy and reliability, the randomized condition was applied to a limited number of participants with possible inter-individual variation in glucose and insulin kinetics. In Experiment 2, we measured the plasma glucose level and IRI after glucose-loading in naïve and experimental subjects with interference of normal air flow or oral taste information in an increased number of participants (n = 7 by adding 3 to the previous 4). Then we compared the data obtained among the three conditions in each participant to decrease inter-individual variation in glucose and insulin kinetics.

The most important finding in the present study is that BGL increase after glucose-loading decreased from trial 1 to trial 2 and trial 3, a third successive oral glucose-loading, produced only negligible increase in BGL, whereas adding an agreeable flavor to the glucose solution returned the BGL to the level found in the first trial. These are novel findings as far as we can ascertain. The three successive oral glucose-loading procedure lasted almost 7 h with the supplement of ingested glucose solution and water, which made the participants more or less bored and tired, especially during the third trial when the taste of the glucose solution no longer seemed palatable or satisfactory, presumably because of the fatigue and sensory-specific satiety (Rolls et al. 1981). Such states can be reflected by increased values for s-AMY, which is a marker of sympathetic nervous activity (Yamaguchi et al. 2004), and as decreased satisfaction scores. More precisely, the first glucose loading was palatable, the second was less palatable and the third loading was not palatable. However, adding an agreeable flavor to the glucose solution rendered the third loading refreshing and palatable (see Table 2). Thus, AUBGC positively correlated with palatability scores and negatively with s-AMY values. Reduced palatability under the uncomfortable state enhances sympathetic nervous activity, indicated by increased s-AMY values. The uncomfortable state might not be so severe because just adding an agreeable flavor to the glucose solution attained the normal increase of BGL level together with increased satisfaction score and decreased s-AMY value.

The relation between stress and gastrointestinal (GI) motility had been demonstrated. Especially the interaction of acute stress with GI motility and chemical mediators including catecholamine and central corticotrophin releasing factor had been shown (Suto et al. 1994; Tsukada et al. 2003). Ochi et al. (2008) examined gastric emptying and chemical mediator at 8 h after onset of stress restraint. They found that continuous stress over 8 h delayed gastric emptying and suggested that delayed gastric emptying at acute phase of continuous stress was mediated via sympathetic nervous system. Thus acute and continuous stress might meditate GI motility followed by glucose absorption from GI tract via ANS.

Furthermore, studies in rats have shown that ingesting food that tastes sweet and bitter respectively enhances and inhibits gastric emptying (Inui-Yamamoto et al. 2009), and that umami (monosodium glutamate) and sweet (sucrose) stimulations of the tongue enhance vagal activities in pancreatic and hepatic branches, whereas 5 % NaCl causes the opposite effects in these nerves (Niijima 1991a, b). These results suggest that the palatability of food enhances gastrointestinal, hepatic, and pancreatic functions, whereas the reverse is true for unpalatable food.

Although no direct measurement of gastric emptying time and ANS function was executed, one interpretation of the decreased BGL after repeated glucose loadings may be that glucose absorption from the GI tract is attenuated because of enhanced sympathetic activity, as shown by increased s-AMY values, when food is unpalatable or rendered unpalatable by uncomfortable conditions.

To further assess the effects of less palatability on the glucose-loading test, we delivered a glucose solution directly into the stomach to eliminate taste and other oral sensations, and clipped the nose during oral glucose-loading to interfere with smooth nasal air-flow and swallowing. The BGL was reduced under both conditions, but eliminating oral sensations tended to exert more powerful effects than nose-clipping. Satisfaction decreased and s-AMY increased in parallel with these reductions, suggesting that glucose intake becomes unpleasant and sympathetic nervous activity becomes activated after such interventions.

Taste stimulation has been shown to be involved in cephalic phase response to food ingestion to influence normal digestive and metabolic processes, e.g., hormone release, gastric acid release, rate of gastric emptying and glucoregulatory mechanism (Feldman and Richardson 1986; Helman 1988; Teff and Engelman 1996; Cecil et al. 1998). Furthermore, intravenous or intragastric glucose administration which is bypassing oral sensory receptors is known to elicit less insulin release than orally ingested glucose via attenuation of the parasympathetic nervous system (Teff and Engelman 1996; Elrick et al. 1964).

## Conclusion

Despite the small sample size of the present study, the results suggest that three successive oral glucose-loadings over 7 h induced a gradual downward shift in the BGC correlated with decreasing satisfaction scores and increasing s-AMY value. However, when a pleasant flavor was added to the glucose solution during the third trial, the BGC was increased to the same level as that during the first loading with decreased s-AMY value, indicating that favorable chemical senses are important in maintaining glucose kinetics. Disrupted normal ingestion during glucose intake modulates glucose kinetics along with increased s-AMY value. To support the present study, direct measurement of ANS function and gastric emptying time in a larger number of participants is the subject for further study.
